# Snoring and stertor are associated with more sleep disturbance than apneas and hypopneas in pediatric SDB

**DOI:** 10.1007/s11325-019-01809-3

**Published:** 2019-03-01

**Authors:** Mark B. Norman, Henley C. Harrison, Karen A. Waters, Colin E. Sullivan

**Affiliations:** 1 Discipline of Sleep Medicine, University of Sydney, Level 4, Chris O’Brien Lifehouse, 119-142 Missenden Road, Camperdown, NSW 2050 Australia; 2 Ear, Nose and Throat Department, Sydney Children’s Hospital, Sydney, Australia; 3 David Read Sleep Laboratory, The Children’s Hospital at Westmead, Westmead, NSW Australia

**Keywords:** Snoring, Stertor, Breath sounds, Auscultation, Partial upper airway obstruction

## Abstract

**Purpose:**

Polysomnography is not recommended for children at home and does not adequately capture partial upper airway obstruction (snoring and stertor), the dominant pathology in pediatric sleep-disordered breathing. New methods are required for assessment. Aims were to assess sleep disruption linked to partial upper airway obstruction and to evaluate unattended Sonomat use in a large group of children at home.

**Methods:**

Children with suspected obstructive sleep apnea (OSA) had a single home-based Sonomat recording (*n* = 231). Quantification of breath sound recordings allowed identification of snoring, stertor, and apneas/hypopneas. Movement signals were used to measure quiescent (sleep) time and sleep disruption.

**Results:**

Successful recordings occurred in 213 (92%) and 113 (53%) had no OSA whereas only 11 (5%) had no partial obstruction. Snore/stertor occurred more frequently (15.3 [5.4, 30.1] events/h) and for a longer total duration (69.9 min [15.7, 140.9]) than obstructive/mixed apneas and hypopneas (0.8 [0.0, 4.7] events/h, 1.2 min [0.0, 8.5]); both *p* < 0.0001. Many non-OSA children had more partial obstruction than those with OSA. Most intervals between snore and stertor runs were < 60 s (79% and 61% respectively), indicating that they occur in clusters. Of 14,145 respiratory-induced movement arousals, 70% were preceded by runs of snore/stertor with the remainder associated with apneas/hypopneas.

**Conclusions:**

Runs of snoring and stertor occur much more frequently than obstructive apneas/hypopneas and are associated with a greater degree of sleep disruption. Children with and without OSA are frequently indistinguishable regarding the amount, frequency, and the degree of sleep disturbance caused by snoring and stertor.

## Introduction

Sleep-disordered breathing (SDB) in children typically presents as a history of snoring, disturbed sleep, and enlarged adenoids/tonsils. An array of daytime symptoms, including attention difficulties and hyperactivity are common [1]. Supervised polysomnography (PSG) is the recommended diagnostic test in clinical practice [2]; however, it is widely recognized that PSG-derived indices do not adequately characterize childhood SDB [3]. While standard PSG metrics report apneas and hypopneas, they do not measure periods of partial upper airway obstruction (UAO) such as snoring that are characteristic of pediatric SDB. Many PSG systems do use snore sensors but the recommended recording parameters [4] only permit capture of a small bandwidth of snore sounds [5–7]. Flow limitation is another sign of UAO seen on PSG but it is not routinely quantified as it is difficult to do so. Additionally, body movements, a robust indicator of sleep disruption intrinsic to actigraphy, are not scored as arousal events in PSG unless there is a concurrent EEG activation [4].

The Sonomat is a non-contact mat system, recently validated against PSG in children for measurement of PSG metrics such as total sleep time (TST) and the apnea/hypopnea index (AHI) [7] with sensitivity and specificity of 86% and 96% respectively at a threshold of 5 events per hour. This system also allows accurate measurement of snoring and stertor [6, 7] which are pathognomonic physical signs of partial UAO and may be associated with sleep disruption [8].

The purpose of this study was to use the Sonomat in the homes of a large group of children with symptoms of SDB and to compare the sleep disturbance (movement arousal) associated with snoring and stertor to that caused by apneas and hypopneas. A secondary aim was to evaluate the unsupervised use of this system when administered by parents/caregivers.

## Materials and methods

This study was undertaken through the David Read Laboratory (University of Sydney, NSW, Australia) with the protocol approved by the University of Sydney Human Research Ethics Committee (HREC Ref: 10–2007/10229). Parents/caregivers of children (< 18 years) with suspected SDB were invited to participate and provided written informed consent if agreeable. Children provided assent when able.

### Participants

Otherwise healthy children (*n* = 231) with a range of symptoms suggestive of SDB, referred clinically to an otolaryngologist for assessment for adenotonsillectomy, were recruited. The parent/caregiver managed a single overnight Sonomat recording in the child’s bed.

The Sonomat (Sonomedical Pty Ltd. Balmain Australia) is placed on the child’s mattress with bed clothes arranged as normal and records breath sounds (similar to a digital stethoscope) and movement without physical attachment of sensors to the body.

**Fig. 1:**
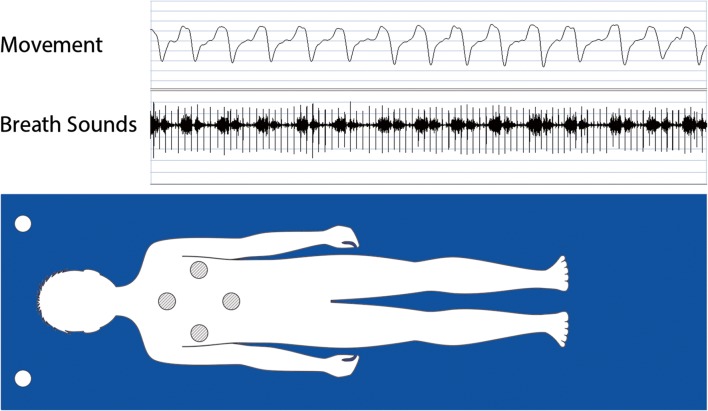
Sonomat sensor location with child lying supine. Four Sonomat sensors (hatched circles) all record sound and movement signals (output from only one sensor shown). Two room sound microphones (open circles) record sound signals

All four Sonomat sensors record the same signals, and this, in conjunction with their physical placement within the Sonomat (Fig. 1), creates a redundancy that permits the child to move around the bed in all directions while minimizing the amount of time off all sensors. Analysis requires signals from only one sensor. Figure 1 shows breathing movements (inspiration up) on the movement channel with breath sounds (first sound = inspiration) and heart sounds (vertical spikes) shown on the breath sounds channel. The breath sounds channel signal can be replayed through audio speakers/headphones and analyzed using spectrographic methods that allow visual display and measurement of the frequency components of the breath sounds.

### Sonomat scoring criteria

Apneas, hypopneas, snoring, stertor, body movements, quiescent time (Qd), poor-quality signals, and instances of the child leaving the bed were scored manually as described in detail previously [7]; however, we include a summary of these scoring criteria. Recordings were scored by MBN and reviewed by CES to ensure scoring consistency.

Briefly, a movement arousal (MA) was identified as an abrupt change in the regular pattern of breathing movements, classified as spontaneous or respiratory-induced based on breath sounds occurring in the 5 s prior to their occurrence. Spontaneous MAs had normal breath sounds preceding whereas respiratory-induced MA events were preceded by apneas, hypopneas, snoring, or stertor. In MA sub-group analysis, examining the specific breathing event preceding respiratory-induced movements, if a hypopnea *and* a run of obstructed breathing occurred simultaneously, the MA was logged as being caused by the hypopnea.

The periods of time between all movements, where the child was quiescent, were considered analogous to sleep and used as the denominator for calculating respiratory event indices.

Snoring was scored when breath sounds contained frequency bands from 20 to ~ 300 Hz and stertor was scored when breath sounds contained no clear frequency peaks but “white noise” from ~ 300 to 2000 Hz. A single breath containing the aforementioned sounds was sufficient to score snore/stertor events. Snoring and stertor are collectively referred to herein as “obstructed breathing”. The term obstructed breathing was chosen as, although snore and stertor each contain different frequency components, both consist of breathing sounds that are pathognomonic indicators of upper airway obstruction.

Apneas were scored when there was an absence of breath sounds and classified based on the presence or absence of breathing movements. Hypopneas were scored when there was a change in the amplitude of the breathing movement channel and further classified based on the presence or absence of obstructed breathing, scored as obstructive and central hypopnea respectively. A minimum of two breath duration was required for scoring of apneas and hypopneas.

### Statistical analysis

Statistical analysis was performed using IBM SPSS Statistics 24 (IBM Corp., Armonk, NY, USA) and GraphPad Prism 7 (GraphPad Software Inc., La Jolla, CA, USA). Normally distributed data are presented as mean ± SEM and non-normally distributed data as median and interquartile range (IQR). The significance of any differences was determined using unpaired *t* tests and Mann Whitney *U* tests and, in multiple measures, was performed using ANOVA for normally distributed data or a Kruskal-Wallace test for non-normally distributed data. Correlation was performed using Spearman’s rank-order method. All tests of significance were two-tailed and a *p* value of < 0.05 was considered significant.

## Results

There were 213 technically successful recordings (92%) from 231 children. The 18 failures comprised no recording in three (17%), < 4 h of interpretable data in seven (39%), and < 4 h lying on the Sonomat in eight (44%); all in this latter group left the bed after several hours of sleep and did not return. Six of the seven children (86%) who were on the Sonomat for ≥ 4 h, but whose recordings contained < 4 h of interpretable data, were ≤ 5 years old. However, although children ≤ 5 years of age predominated in this group of poor-quality recordings, they were only a small proportion (5%) of the 129 children in this age group.

**Fig. 2:**
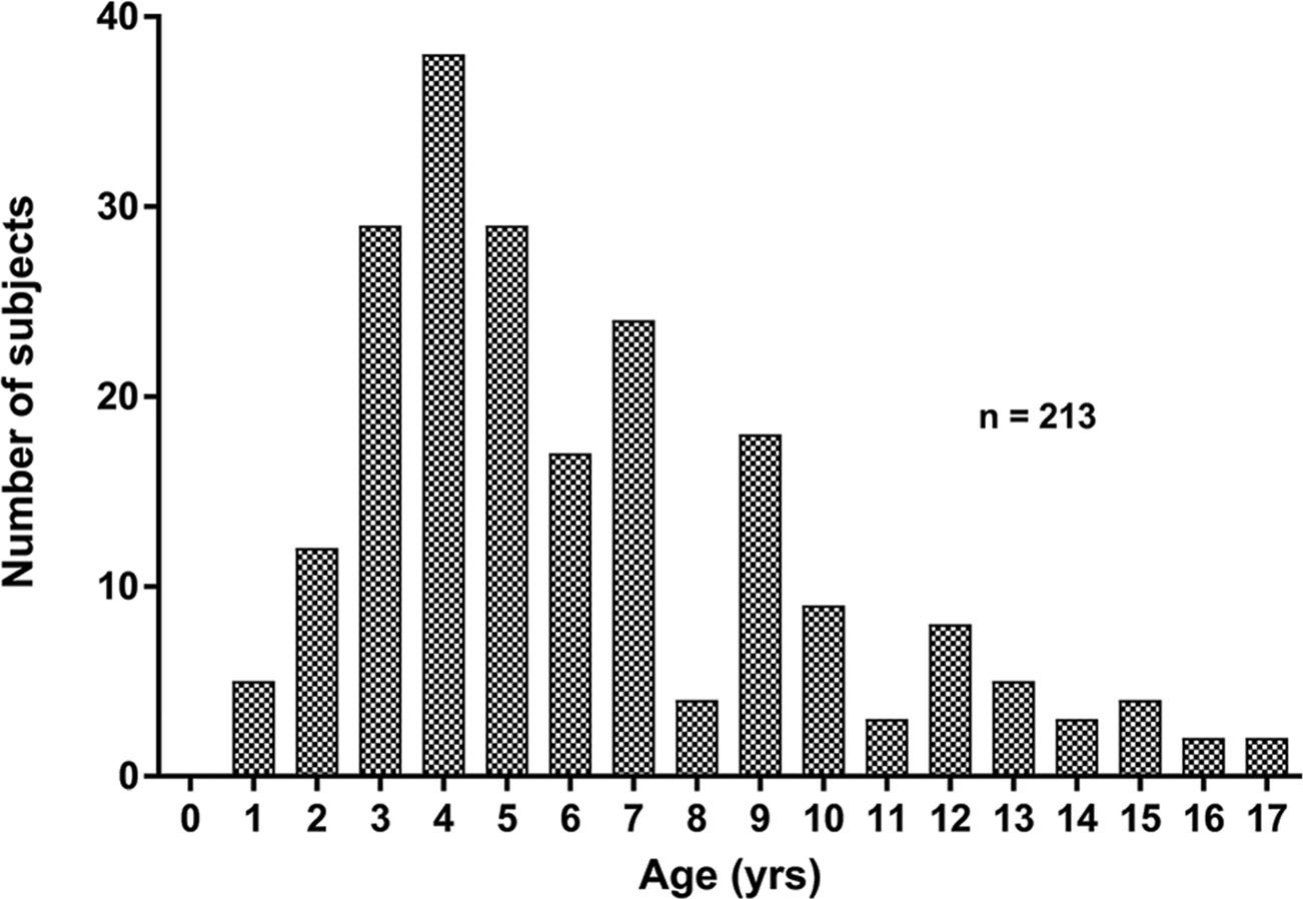
Frequency distribution of the ages of children studied

The cohort was aged 5.2 years (3.7, 8.5) range 0.8–17.7, 120 (56%) were male, with BMI z-score = 0.46 (− 0.35, 1.7) (*n* = 154 with height/weight information). Two-thirds of children (*n* = 141) were ≤ 6 years (Fig. 2).

There were 116,605 min recorded (range 284 to 733) with uninterpretable signals for 10,841 min (9% of TRT) leaving 105,764 min (91% of TRT) available for analysis (range 240 to 710). Uninterpretable signals included poor-quality signals (max = 382 min) and time out of bed (max = 250 min). There were 145 children (68%) with ≥ 1 episode of poor signal quality and 59 (28%) left their bed on ≥ 1 occasion. Detailed time and movement data are shown in Table 1.

**Table 1:** Recording time, body movement, and quiescent time data

Variable	Duration (min)	Percentage	Number (/h)
TRT	557.1 (496.6, 604.5)	–	–
Poor signal quality	16.4 (0.0, 69.0)	3.4 (0.0, 12.0)	–
Out of bed	0.0 (0.0, 0.7)	0.0 (0.0, 0.1)	–
Analysis time	513.6 (425.9, 573.5)	95.7 (85.7, 100.0)	–
Quiescent time (Qd)	474.0 (391.1, 532.6)	93.4 (91.6, 94.7)^a^	–
Spont. MA	23.1 (16.4, 31.4)	4.8 (3.6, 6.4)	13.7 (10.1, 17.8)
Resp. MA	5.6 (2.2, 14.3)	1.3 (0.4, 3.0)	4.5 (1.4, 12.3)
Total MA	31.9 (24.8, 41.0)	6.6 (5.3, 8.4)	21.0 (16.0, 28.5)

Values are median (interquartile range)

*TRT* total recording time, *Qd* quiescent time, *Spont.* spontaneous, *MA* movement arousal, *Resp.* respiratory-induced

^a^Analogous to sleep efficiency

### Apnea and hypopnea events

Obstructive apneas were present in 114 (54%), central apneas in 182 (85%), and mixed apneas in 103 (48%) children. Obstructive hypopneas were present in 136 (64%) and central hypopneas in 116 (54%). There were 13 children (6%) with no apneas or hypopneas.

### Snore and stertor events

Snore runs were present in 201 (94%), runs of stertor in 132 (62%) and, combined, obstructed breathing was present in 202 (95%). There were 11 children (5%) with no evidence of UAO. Over half the snoring children also had stertor (65%) whereas 99% of stertorous children snored.

Table 2 details the occurrence of apneic, hypopneic, and obstructed breathing events.

**Table 2:** Presence and duration of SDB pathology

	No.	Occurrence (/h)	Duration (s)	Range (s)	Total duration (min)	Duration (% of Qd)
All resp. events^a^	8735	2.3 (0.8, 6.3)	12.0 (9.0, 17.0)	1.8–77.2	1998	2.0
OA	1955	0.1 (0.0, 0.9)	9.0 (7.0, 12.0)	2.0–53.0	331	0.3
CA	1497	0.5 (0.2, 1.1)	9.0 (7.0, 11.0)	3.0–77.2	229	0.2
MA	445	0.0 (0.0, 0.4)	11.0 (8.7, 13.0)	3.4–40.0	84	0.1
All apneas	3897	1.1 (0.4, 2.7)	9.0 (7.0, 12.0)	2.0–77.2	645	0.7
OH	4263	0.4 (0.0, 2.8)	15.0 (12.0, 20.2)	1.8–72.0	1209	1.2
CH	575	0.1 (0.0, 0.4)	13.0 (10.0, 17.7)	3.0–55.0	144	0.1
All hypopneas	4838	0.8 (0.1, 3.7)	15.0 (11.1, 20.0)	1.8–72.0	1353	1.4
Obstructive Events ^b^	6663	0.8 (0.0, 4.7)	13.0 (9.0, 18.0)	1.8–72.0	1625	1.7
Central events ^c^	2072	0.8 (0.4, 1.8)	10.0 (7.0, 12.9)	3.0–77.2	373	0.4
Snore	28,420	14.5 (5.0, 27.4)	10.5 (4.1, 28.0)	0.6–3595	18,115	18.2
Stertor	3852	0.3 (0.0, 3.2)	12.2 (6.0, 28.7)	0.6–2338	2381	2.4
Obstructed breathing ^d^	32,272	15.9 (5.3, 30.1)	11.0 (4.8, 28.0)	0.6–3595	20,508	20.6

Values are median (interquartile range)

*No.* number, *Qd* quiescent time, *OA* obstructive apnea, *CA* central apnea, *MA* mixed apnea, *OH* obstructive hypopnea, *CH* central hypopnea

^a^Apneas + hypopneas

^b^OA + MA + OH

^c^CA + CH

^d^Snore + stertor

### Obstructive events

There were 38,935 obstructive upper airway events (obstructive apneas, mixed apneas, obstructive hypopneas, and obstructed breathing runs). Of these, 17% were apneas/hypopnea events and 83% were obstructed breathing events. Table 2 shows a comparison of these MOAHI and obstructed breathing events in detail.

**Table 3:** Differences in time and movement variables across different age ranges

	≤ 2 years	3–5 years	6–8 years	9–12 years	≥ 13 years	*p* value
Subjects, *n*	34	89	47	29	14	–
TRT, min	544.7 ± 14.4	540.5 ± 9.9	560.6 ± 11.3	563.9 ± 8.5	522.8 ± 13.5	0.356
Poor signal quality, min	72.7 (7.2, 170.0)	21.0 (0.0, 69.0)	27.5 (0.0, 69.7)	1.3 (0.0, 15.6)	0.0 (0.0, 50.0)	< 0.0001
Out of bed, min	0.0 (0.0, 2.3)	0.0 (0.0, 1.6)	0.0 (0.0, 0.0)	0.0 (0.0, 0.3)	0.0 (0.0, 0.0)	0.046
Analysis time, min	442.4 ± 14.9	489.5 ± 11.3	514.8 ± 15.4	549.2 ± 8.8	493.1 ± 16.7	0.0003
Total MA index, /h	31.5 (25.9, 38.0)	20.7 (16.8, 28.3)	20.2 (15.0, 24.9)	16.5 (13.8, 20.6)	14.0 (11.7, 17.4)	< 0.0001
Total MA time, %	8.3 (6.8, 10.6)	6.4 (5.1, 8.4)	6.7 (5.7, 8.5)	5.5 (4.5, 6.8)	5.3 (4.6, 6.6)	< 0.0001
Spont. MA index, /hr	14.1 (10.7, 21.2)	15.5 (11.7, 18.8)	12.8 (7.9, 17.3)	12.1 (8.6, 14.9)	11.3 (8.9, 15.8)	0.0231
Spont MA time, %	5.8 (3.5, 7.1)	5.1 (4.0, 6.4)	4.8 (3.1, 6.5)	4.1 (3.2, 5.3)	4.6 (3.1, 6.1)	0.142
Resp. MA index, /hr	15.4 (7.4, 22.3)	3.6 (1.3, 10.4)	3.9 (1.5, 11.5)	4.0 (1.0, 9.5)	2.5 (1.0, 5.7)	< 0.0001
Resp. MA time, %	3.0 (1.5, 4.2)	1.1 (0.4, 3.0)	1.1 (0.4, 3.0)	1.3 (0.2, 2.3)	0.7 (0.2, 1.4)	0.0002
Qd, min	402.5 ± 14.3	456.1 ± 10.7	478.7 ± 14.4	517.3 ± 7.9	465.4 ± 16.0	< 0.0001

Values are median (interquartile range) and mean ± SEM where appropriate

*TRT* total recording time, *MA* movement arousal, *Spont.* spontaneous, *Resp.* respiratory-induced, *Qd* quiescent time

### Age-related differences

Different age ranges were examined in relation to time and movement (Table 3) in addition to respiratory variables (Table 4). Poor signal quality, time spent out of bed, respiratory-induced MAs, spontaneous MA frequency, obstructed breathing, the AHI, and the MOAHI all decreased with increasing age whereas analysis time and Qd tended to increase with age. Parameters that did not change with age were TRT and spontaneous MA duration. There was a weak relationship between age and the MOAHI (r = − 0.181, *p* = 0.008) with younger children tending to higher values. Similarly, moderate relationships existed between age and snore frequency (r = − 0.319, *p* < 0.0001) and duration (r = − 0.162 *p* = 0.018) with younger children snoring more often and for longer. There was a moderate decrease in percent time moving as age increased (r = − 0.382; *p* < 0.0001) but a strong decrease in the frequency of MAs as age increased (r = − 0.511; *p* < 0.0001).

**Table 4:** Differences in respiratory variables across different age ranges

	≤ 2 years	3–5 years	6–8 years	9–12 years	≥ 13 years	*p* value
Subjects, n	34	89	47	29	14	–
Snoring, %	27.9 (14.6, 42.4)	13.2 (3.2, 21.7)	14.1 (2.1, 31.7)	7.1 (0.5, 26.0)	7.9 (0.7, 18.8)	0.001
Snore runs, /h	27.8 (17.5, 30.4)	12.6 (3.6, 27.0)	14.5 (5.8, 26.0)	6.2 (2.2, 22.1)	9.3 (1.6, 14.3)	< 0.0001
Stertor, %	2.2 (0.1, 4.1)	0.0 (0.0, 2.0)	0.0 (0.0, 3.9)	0.0 (0.0, 0.9)	0.0 (0.0, 0.7)	0.0073
Stertor runs, /h	2.7 (0.6, 5.9)	0.2 (0.0, 3.2)	0.2 (0.0, 3.1)	0.0 (0.0, 1.7)	0.0 (0.0, 0.8)	0.0007
OB, %	30.4 (16.8, 50.1)	14.4 (3.4, 28.7)	16.0 (2.3, 40.8)	7.1 (0.7, 26.5)	8.9 (1.3, 18.8)	0.0011
OB runs, /h	30.8 (22.2, 36.6)	14.1 (3.9, 30.3)	15.3 (5.8, 28.2)	6.7 (3.0, 23.1)	9.5 (3.3, 14.4)	< 0.0001
AHI, events/h	5.5 (3.3, 16.7)	1.7 (0.8, 5.6)	2.0 (0.5, 6.5)	2.0 (0.8, 5.3)	1.4 (0.8, 2.7)	0.0036
MOAHI, events/h	3.5 (1.6, 11.5)	0.4 (0.0, 4.2)	0.7 (0.0, 5.1)	1.1 (0.0, 3.1)	0.5 (0.0, 1.3)	0.0011

Values are median (interquartile range) and mean ± SEM where appropriate

*OB* obstructed breathing, *AHI* apnea/hypopnea index, *MOAHI* mixed and obstructive apnea/hypopnea index

### OSA versus non-OSA children

Using standard OSA severity criteria 113 (53.1%) children were normal (MOAHI < 1 events/h), 49 (23.0%) had mild OSA (1 ≤ MOAHI < 5 events/h), 26 (12.2%) had moderate OSA (5 ≤ MOAHI < 10 events/h), and 25 (11.7%) had severe OSA (MOAHI ≥ 10 events/h); there were 100 (46.9%) OSA children with 51 (23.9%) of these classified as moderate to severe.

**Fig. 3:**
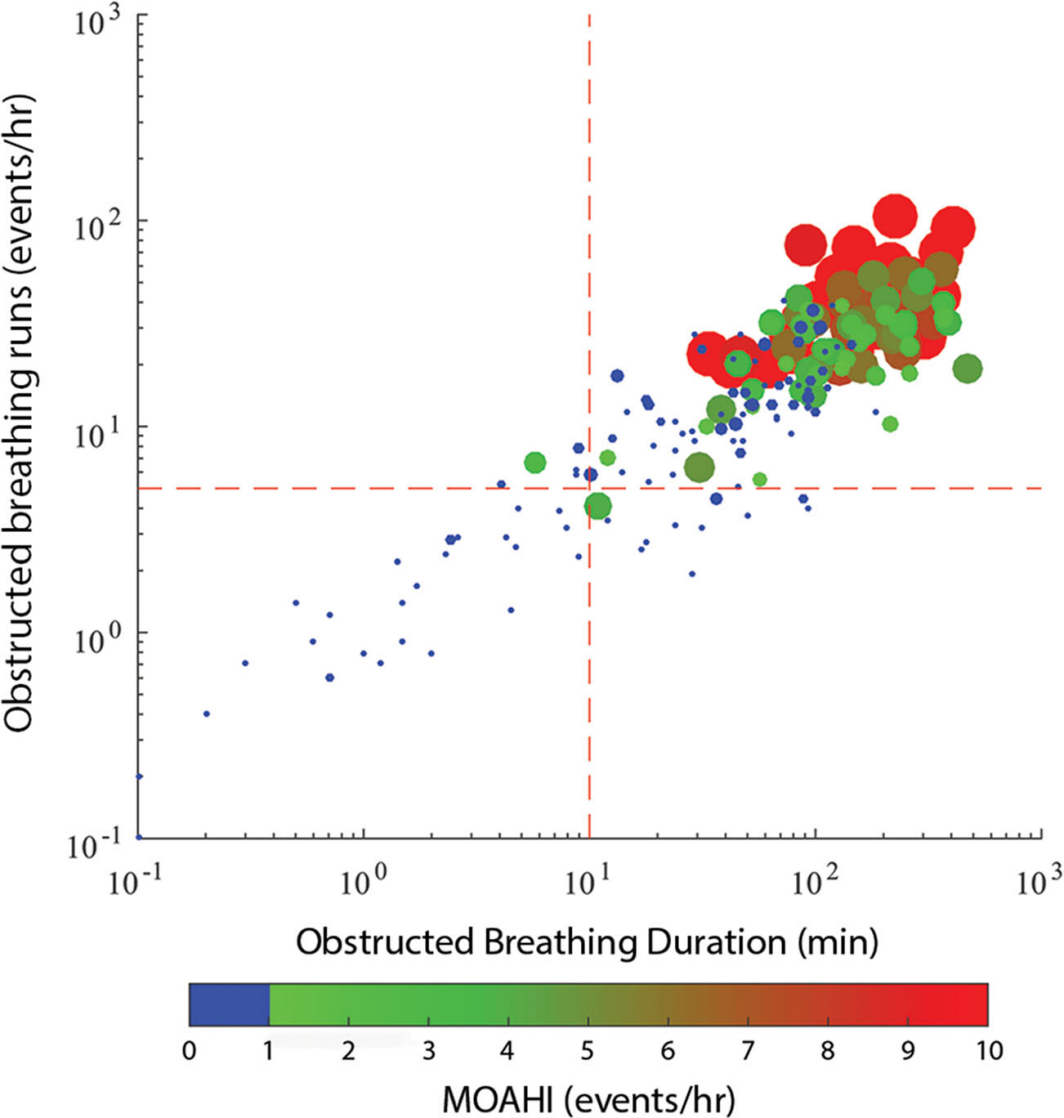
Bubble plot showing total duration (log_10_, x-axis) plotted against number of runs (log_10_, y-axis) of obstructed breathing for each child. MOAHI values are on the z-axis; blue circles are non-OSA children with hotter colors and larger circles indicating increasing severity of OSA (all MOAHI values ≥ 10 events/h have the same diameter). Red dashed lines indicate thresholds of 10 min (x-axis) and 5 events/h (y-axis). *MOAHI* mixed and obstructive apnea/hypopnea index

OSA children had longer obstructed breathing duration (OSA = 140.9 [86.6, 225.6], non-OSA = 23.3 [1.6, 61.2] min; *p* < 0.0001) and more obstructed breathing runs (OSA = 30.7 [19.9, 39.6], non-OSA = 6.1 [1.4, 13.7] runs/h; *p* < 0.0001); individually, snore and stertor were also significantly greater in OSA children. However, long periods (≥ 10 min) of obstructed breathing occurred in 69 (61.1%) and frequent runs (≥ 5/h) of obstructed breathing occurred in 63 (55.8%) of the 113 non-OSA children (Fig. 3).

In the non-OSA group (*n* = 113, 59 male), 12 (10.6%) had no snoring, 11 (9.7%) snored for < 1.0 min, 16 (14.2%) snored for ≥ 1.0–4.9 min, 6 (5.3%) snored for ≥ 5.0–9.9 min, and 68 (60.2%) snored for ≥ 10 min. The 68 non-OSA snoring children spent 48.3 min (24.6, 82.7) (range 12.5–185.2) snoring, at a rate of 12.1 (8.5, 18.3) snore runs/h (range 1.9–39.4). In this same non-OSA group, 75 (66.4%) had no stertor, 24 (21.2%) had stertor present for < 1.0 min, 7 (6.2%) had stertor present for 1.0–4.9 min, 1 (0.9%) had stertor present for ≥ 5.0–9.9 min, and 6 (5.3%) had stertor present for ≥ 10 min. The 6 non-OSA stertorous children had 21.6 min (12.9, 36.2) of stertor (range 10.0–42.3) occurring at a rate of 1.5 (0.4, 2.9) stertor runs/h (range 0.4–4.3).

Runs of obstructed breathing were frequent and brief (Table 5) with the interval between the end of one run and the beginning of the next demonstrating their fragmentary nature (Table 6). There were 28,220 intervals between snore runs and 3722 between stertor runs; the duration of these intervals differed significantly (snore = 17 [9, 47], stertor = 31 s [10, 201], *p* < 0.0001). Only 8.6% of the intervals between snore runs were ≥ 5 min (300 s) whereas 21.4% of intervals between stertor runs were ≥ 5 min. Two-thirds (65.7%) of snore runs and half (49.1%) of stertor runs occurred < 30s apart.

**Table 5:** Comparison of usual MOAHI events and obstructed breathing events

	Obstructive/mixed apneas and hypopneas	Obstructed breathing	*p* value
Occurrence (/h)	0.8 (0.0, 4.7)	15.9 (5.3, 30.5)	< 0.0001
Duration of event (s)^a^	13.0 (9.0, 18.0)	11.0 (4.8, 28.0)	< 0.0001
Total duration (min)^b^	1.2 (0.0, 8.5)	69.9 (15.7, 140.9)	< 0.0001

Values are median (interquartile range)

^a^Median duration of individual events

^b^Total duration of all events

**Table 6:** Time intervals between runs of snore and stertor

Interval (s)	< 10	< 30	< 60	< 120	< 300	< 600	> 600
Snore (*n*)	8080	18,554	22,164	24,197	25,796	26,685	1535
Percent of all snore intervals (%)	28.6	65.7	78.5	85.7	91.4	94.6	5.4
Stertor (*n*)	857	1828	2259	2594	2927	3132	590
Percent of all stertor intervals (%)	23.0	49.1	60.7	69.7	78.6	84.1	15.9

As obstructed breathing *duration* increased, the % time moving and frequency of movements increased (r = 0.219, *p* = 0.0013 and r = 0.396, *p* < 0.001 respectively). As obstructed breathing *frequency* increased, both the % time moving and frequency of movements increased (r = 0.229, *p* = 0.0008 and r = 0.540, *p* < 0.0001 respectively). Positive correlations were also present between the MOAHI and % time moving (r = 0.263, *p* = 0.0001) and movement frequency (r = 0.548, *p* < 0.0001). Thus, indices of obstructed breathing and the MOAHI all correlated to a similar degree with sleep disruption.

### Respiratory event-induced movement arousals

Subgroup analysis of movement arousals showed that 4248 (48.6%) of 8735 apneas and hypopneas were terminated with a body movement compared to 9897 (30.7%) of 32,272 obstructed breathing runs. However, while apneas and hypopneas were more likely to be associated with a MA, most (9897 or 70.0%) of the 14,145 MAs were preceded by obstructed breathing.

## Discussion

While it has long been recognized that childhood SDB is characterized by “prolonged partial upper airway obstruction” [9], objective measurement has proven elusive. Various methods have attempted to compensate for the limitations of AHI metrics [3], but their invasive [10] or complex [11] nature has precluded widespread acceptance in clinical practice. Quantitative measurement of snoring and stertor with the Sonomat system provides a practical way of assessing partial UAO, in addition to identifying discrete apnea/hypopnea events. The failure rate in an unsupervised environment was very low, confirming that the Sonomat is practical and easy to use with children in the home.

A small proportion of children, not unexpectedly, left their bed and did not return (possibly to sleep in their parent’s bed). Those whose studies failed due to poor signal quality tended to be younger (≤ 5 years). It is possible that their small size allowed them to lie between the four sensors for long periods although one study was failed due to the presence of a cooling fan masking signals from all sensors. Despite children ≤ 5 years of age predominating in the studies deemed to have failed due to poor-quality signals, they were only a small proportion of all children aged 5 years or younger. Additionally, although we show that the duration of poor-quality signal is greater in younger age groups, the duration of good quality recordings remains at > 7 h (Table 3). Therefore, a younger age does not contraindicate Sonomat use. In our experience, younger children are ideally suited for Sonomat use as they are often the most distressed during polysomnography.

Our results in this large group extends previous work in which the method was validated against PSG [7] and confirms that obstructed breathing predominates as an indicator of pediatric SDB. Quantification of obstructed breathing identified 98% of OSA children with only 2 children crossing one, but not both obstructed breathing thresholds (Fig. 3). Thus, the absence of obstructed breathing could be used to discount the presence of OSA. Most importantly, OSA and non-OSA children were frequently indistinguishable regarding the frequency and duration of obstructed breathing and many symptomatic children did not have OSA or obstructed breathing.

A major new finding is that runs of snoring and stertor are linked to most movement arousals resulting in a level of sleep disruption that is an order of magnitude greater than that associated with apneas/hypopneas alone. The current standard metrics generated from routine PSG, focusing on apneas and hypopneas, underestimate the nature and extent of partial UAO in children and its major role in sleep disruption.

The finding that obstructed breathing runs are frequently terminated by movement may help to explain why some robust measures of SDB outcome do not have a simple relationship with the AHI. The most notable is that SDB of any severity, including “simple” snoring, is associated with elevated blood pressure [12]. Our data support the idea that obstructed breathing, not just AHI-linked events, is a major pathophysiological pathway in pediatric SDB. We suggest that the further step of providing an accurate measurement of obstructed breathing, as a complementary measurement to AHI metrics, will further clarify links to cardiovascular and other outcomes.

Our analyses show that snore and stertor runs are brief and, as they are also separated by short intervals, tend to occur in clusters. Without an electroencephalogram (EEG), we cannot confirm that the episodes of normal breathing between obstructed breathing runs occur during wake/arousal or sleep; however, they would undoubtedly have a fragmentary effect on sleep and/or sleep processes. Even in the absence of a MA, a change from abnormal to normal breathing strongly indicates that some form of activation has occurred within the airway. Work investigating respiratory effort-related arousal events [13] would support this.

These data may also provide an explanation for anomalies in the landmark CHAT study [14, 15]. The first anomaly was a lack of correlation between the AHI and clinical measures of severity from the pediatric sleep questionnaire (PSQ). Secondly, the AHI did not predict the response to surgery, whereas the PSQ did. At face value, these results question the utility of PSG. Our findings, that large numbers of obstructed breathing runs are associated with sleep disruption, could explain this as partial UAO was not quantified in the CHAT study.

Our results also align with recent prospective work identifying adverse impacts of childhood SDB in cognitive and behavioral domains. Hunter et al. [16] found an association between SDB and cognitive dysfunction that was particularly marked in moderate/severe OSA. In contrast, separate analysis in the same cohort [17] found that behavioral dysfunction was most prominent in the mildest SDB (habitually snoring children without OSA). Furthermore, abnormalities in the behavioral domain were less marked as SDB severity increased. In these two analyses, the history of snoring (assessed by questionnaire) was included with the AHI as the independent variable. More recently, the same data was re-analyzed with snoring and OSA assessed as separate variables [18]. It emerged that the frequency of snoring (parentally reported weekly frequency, not to be confused with frequencies contained within snoring sounds) was better correlated with behavioral dysfunction than was the AHI. Additionally, while behavioral abnormalities were less marked as the AHI increased, abnormalities in cognitive measures were worse. Notably, these relationships were apparent despite snoring “frequency” being assessed only by parental reports. The findings are consistent with the interpretation that sleep fragmentation induced by runs of snoring/stertor may have the greatest impact on behavior, whereas changes in blood gases associated with apneas/hypopneas may have a more pronounced effect on cognition.

The two dominant pathophysiological pathways in SDB, sleep fragmentation and blood gas changes, both entrain a variety of different and overlapping downstream effects. Blood gas changes induce major reflex responses (hypertension and arousal) as well as more direct adaptive and maladaptive cellular changes [19]. Arousal and sleep fragmentation induces sympathetic activation (which may contribute to hypertension) and impacts on sleep-dependent brain function [20]. It is not surprising that SDB can produce arousal without significant blood gas changes. There is substantial data implicating that a major pathway to arousal from sleep are upper airway reflexes induced by vibratory stimuli [21]. We suggest that obstructed breathing is a sensitive and specific indicator of pediatric SDB that will show strong correlations with a different spectrum of objective outcomes than does the MOAHI alone [22].

Negative intrathoracic and increased intracranial pressures may be another set of important pathophysiological pathways in SDB; both are known to be influenced by UAO [23]. The longer the duration that abnormal pressures are present may be important and the total duration of UAO may be a useful measurement to indicate this. The total duration of UAO is not typically measured in PSG but we show that obstructed breathing occurs for 60 times longer than do obstructive apneas/hypopneas in this group of symptomatic children (Table 5). Prolonged partial obstruction is a key mechanism of elevated carbon dioxide levels, and in turn, a driver of cerebrospinal fluid pressures and sympathetic outflow. Future work examining the relationship between time spent in obstructed breathing and various outcomes is clearly warranted.

These subjects were typical of a clinical group of children referred to an otolaryngologist needing assessment for adenotonsillectomy. All had a range of symptoms indicative of SDB, for which the primary care physician thought assessment was required and is representative of the ~ 90% of children who undergo adenotonsillectomy without PSG evaluation [24, 25]. We incidentally found nocturnal asthma in one child. Although some snoring was present, the major component of this child’s disordered breathing during sleep consisted of wheeze. This exemplifies work showing that parents are unreliable in both determining the severity of OSA [26] and in identifying wheeze [27] and has important clinical implications.

Limitations include the lack of an EEG to identify sleep and arousal. However, previous work confirmed a close relationship between Qd and EEG-defined sleep as well as accurate calculation of MOAHI [7] and the use of body movement is a robust indicator of sleep disturbance [28]. Adding oximetry to the Sonomat system provides a more complete evaluation of SDB but requires attachment to the body; we have experienced problems using this additional channel, particularly in the home. Transcutaneous carbon dioxide measurement has the same physical constraints although it may be a better indicator of the downstream effects of obstructed breathing [29].

## Conclusion

The Sonomat is a reliable method for quantifying and characterizing the extent and disruptive nature of partial UAO in children in the home environment as well as measuring the standard metrics of SDB. Our previous work has been extended in this study by linking runs of snoring and stertor with sleep fragmentation to an extent that is an order of magnitude greater than that caused by apnea/hypopnea events. Thus, many children classified as “simple” snorers without OSA may have numerous runs of snoring and/or stertor that fragment sleep. These findings may help to explain apparent anomalies between the downstream effects of SDB and measurements obtained using standard PSG. Finally, many children who would progress to surgery on clinical indicators, without objective measurement of SDB, may have minimal partial obstruction and no obstructive respiratory events.
